# One-year follow-up of patients with hearing impairment following traumatic brain injury

**Published:** 2026-01

**Authors:** Reza Fatahian, Mohammad Abdullahzadeh, Masoud Sadeghi

**Affiliations:** ^*a*^ Department of Neurosurgery, School of Medicine, Kermanshah University of Medical Sciences, Kermanshah, Iran.; ^*b*^ Student Research Committee, Kermanshah University of Medical Sciences, Kermanshah, Iran.; ^*c*^ Medical Biology Research Center, Health Technology Institute, Kermanshah University of Medical Sciences, Kermanshah, Iran.

**Keywords:** Traumatic Brain Injuries, Hearing Loss, Sensorineural Hearing Loss, Temporal Bone Fractures, Glasgow Coma Scale

## Abstract

**Background:**

Hearing impairment is a potential consequence of brain trauma and may present as conductive, sensorineural, or mixed hearing loss.

**Methods:**

This descriptive-analytical study included patients who sustained brain trauma between 2018 and 2019 and reported hearing difficulties. All participants were examined by an ENT specialist, and audiometric evaluations were conducted immediately after trauma and again one year later. Based on inclusion criteria, all patients referred to Ayatollah Taleghani medical centers were enrolled, resulting in a sample of 70 cases. Data were analyzed using SPSS version 22, with chi-square tests applied at a significance level of p = 0.05.

**Results:**

The mean age of participants was 33 ± 18 years, with males comprising 84% of the sample. A significant correlation was observed between initial and one-year follow-up audiometric findings. Among patients with temporal bone fractures, 60% exhibited sensorineural and conductive hearing loss.

**Conclusions:**

Sensorineural hearing loss was identified as the most prevalent disorder, particularly associated with moderate trauma (GCS scores 9–12). Temporal bone fractures, primarily resulting from accidents, were the most common type of brain injury, with 48.5% of cases showing right-sided involvement.

## Introduction

Traumatic brain injury (TBI) is a major socio-economic problem worldwide, causing significant death and disability among young and active populations.^1,2^ Its leading causes differ by region: traffic accidents dominate in developing countries, while falls and violence are increasingly important in developed nations.^1^ The incidence of TBI requiring hospitalization in Europe and Australia is reported at 235 per 100,000 people, with most patients being young, thereby imposing heavy social and economic burdens.^2^ TBI results from external forces such as direct blows, rapid acceleration, penetrating injuries, or blast exposure, and its severity depends on the nature and intensity of these forces.^3^ Defined by the International Brain Injury Association as brain damage caused by external force, TBI can lead to temporary or permanent physical, cognitive, and behavioral impairments,^4^ and is a major cause of long-term disability in individuals under 45.^5^ Globally, its burden is substantial: in the U.S., 1.4 million cases occur annually, with 50,000 deaths,^6,7^ while in Iran, head trauma ranks second in mortality, with 55,428 cases reported in Tehran between 2011–2012, mostly among men.^8-10^

The prefrontal cortex is particularly vulnerable, leading to deficits in executive functions such as planning, memory, attention, and hearing.^11^ TBI is classified into primary injuries—caused directly by external forces—and secondary injuries, which develop later through mechanisms such as neurotransmitter release, oxidative stress, and inflammation^12,13^. Hearing loss, especially sensorineural, is a recognized complication of TBI and may result from damage to peripheral or central auditory systems. While traditionally thought to resolve spontaneously, recent evidence shows persistent symptoms resembling post-concussive syndrome, with hearing complaints lasting years or even decades.^14,15^ The exact mechanisms remain unclear, as deficits may stem from cognitive impairments rather than purely auditory dysfunction.^16^ Nonetheless, several studies have reported hearing impairment following TBI.^17-19^ This study therefore aims to investigate the impact of traumatic brain injury on hearing function and explore ways to improve quality of life in affected patients.

## Material and Methods 

**Study Population and Sampling**

This descriptive-analytical study was conducted following approval from the Ethics Committee and the Vice President for Research at Kermanshah University of Medical Sciences, in Ayatollah Taleghani Hospitals. The study population included all patients who sustained brain trauma between 2018 and 2019 and subsequently reported hearing problems. Each patient was examined by an ENT specialist and underwent audiometric testing. The onset of hearing impairment was determined based on the patient’s initial history, and audiometric findings were assessed both immediately after trauma and one year later. Patient information was extracted from medical records, and due to the low prevalence of this condition, all eligible patients referred to Ayatollah Taleghani medical centers during the study period were included (census sampling). In total, 70 patients were evaluated.

**Criteria**

**Inclusion Criteria: **Patients with hearing loss following trauma, confirmed through initial complaints, neurological examination, and audiometry. Exclusion Criteria: Patients with a history of hearing loss prior to trauma, or those unable to communicate or cooperate, were excluded.

**Study Design and Implementation**

 Data collection was carried out at Ayatollah Taleghani Hospitals using a checklist prepared by the project manager, which included the study’s main variables. Sampling was performed as a census. In cases where medical records were incomplete, missing information was obtained by contacting patients via phone. If contact was not possible and essential data were unavailable, the patient’s file was excluded. Final data were entered into Excel and subsequently analyzed using statistical software.

**Data Analysis**

 All data were processed using SPSS version 22. Statistical analysis included chi-square tests for qualitative variables and T-tests for quantitative variables. When data did not follow a normal distribution, appropriate non-parametric tests were applied. A p-value of 0.05 was considered the threshold for statistical significance.

## Results

In this study, 70 brain trauma patients with a mean age of 33 ± 18 years and hearing impairment who had referred to Ayatollah Taleghani Medical Center in Kermanshah were included and examined (Table 1).

**Table 1:** Descriptive characteristics of the variables under study.

Variable Name	Number	Percentage (%)
**Gender**		
Male	59	84.3
Female	11	15.7
**Types of Trauma**		
Fall	8	11.4
Accident	50	71.4
Conflict	12	17.2
**Autotrauma**		
Yes	44	62.9
No	26	37.1
**Side Involved**		
Right	34	48.5
Left	26	37.2
Both Sides	10	14.3

Table 2 shows the frequency of various types of trauma, traumatic brain injury findings, and related clinical manifestations stratified by gender in the study population (59 males and 11 females). Among the types of trauma, accidental injuries were the most prevalent in both males (73.0 %) and females (63.6 %), followed by falls and conflict-related trauma, each accounting for approximately 13–18 % across genders. In terms of brain injury findings, over half of male participants (52.5 %) had normal imaging, whereas this was true for a smaller proportion of females (27.2 %). Among abnormal findings, temporal linear fractures (15.2 % in males, 9.1 % in females) and contusions (8.5 % and 9.1 %, respectively) were relatively common. Other injury types such as epidural and subdural hemorrhages, brain edema, skull base fractures, and subarachnoid hemorrhage were observed at lower frequencies, with several of these showing higher percentages in females, though the female sample was small. Pneumocephalus was seen only in males (5.1 %). Otorrhagia was reported in a majority of both males (61.0 %) and females (63.6 %), whereas otorrhea was less common but proportionally more frequent in females (27.2 %) than in males (10.2 %). Dizziness was relatively uncommon, occurring in 6.8 % of males and 9.1 % of females. Taken together, these findings indicate that while accidental trauma and normal brain imaging findings are common in both genders, various types of intracranial injuries and related symptoms show gender-specific distribution patterns in this study.

**Table 2:** Determining the frequency of types of trauma, the presence of types of traumatic brain injuries, and conductive disorders by gender.

Variable	Male (n, %) (N =59)	Female (n, %) (N= 11)
**Types of Trauma**		
Fall	8 (13.5%)	2 (18.2%)
Accident	43 (73%)	7 (63.6%)
Conflict	8 (13.5%)	2 (18.2%)
**Brain Injuries**		
Epidural	3 (5.1%)	2 (18.2%)
Subdural	3 (5.1%)	1 (9.1%)
Contusion	5 (8.5%)	1 (9.1%)
Normal	31 (52.5%)	3 (27.2%)
Brain Edema	2 (3.4%)	1 (9.1%)
Temporal Linear Fx	9 (15.2%)	1 (9.1%)
Skull Base Fx	1 (1.7%)	1 (9.1%)
SAH	2 (3.4%)	1 (9.1%)
Pneumocephalus	3 (5.1%)	–
**Otorrhagia**		
Yes	36 (61%)	7 (63.6%)
No	23 (38.9%)	4 (36.4%)
**Otorrhea**		
Yes	6 (10.2%)	3 (27.2%)
No	53 (89.8%)	8 (72.8%)
**Dizziness**		
Yes	4 (6.8%)	1 (9.1%)
No	55 (93.2%)	10 (90.9%)

The combined analysis highlights significant associations between traumatic brain injury, hearing impairment, and patient consciousness levels (Table 3). The Wilcoxon test revealed a statistically significant change in hearing impairment over time (p = 0.03), confirming that auditory deficits persisted or evolved during follow-up. Examination of trauma types against Glasgow Coma Scale (GCS) scores showed that accidents were the predominant cause across all levels of consciousness, with 85% of severe cases (GCS 8–3) linked to accidents (p <0.001), and similar dominance observed in moderate and mild cases (p <0.05). Furthermore, the relationship between trauma type and hearing impairment demonstrated that most accident-related cases had normal hearing (72%), though conductive hearing loss (18%) was also notable, while falls and conflicts produced more varied outcomes including sensorineural and mixed losses (p <0.05). Overall, accidents were the leading cause both severe brain injury and hearing complications, underscoring their critical role in patient outcomes.

**Table 3:** Statistical Analysis of Hearing Impairment, Trauma Type, and Level of Consciousness in Patients with Brain Injury.

Analysis	Category	Details	Findings	P-value
Wilcoxon Test (Follow-up Hearing Impairment)	Initial vs. one-year audiometry	Average rating: 3; Total ranks: 15	Significant change in hearing impairment over time	0.03
Relationship between GCS Level and Trauma Type	Severe (GCS 8–3)	Fall: –; Quarrel: 1 (14%); Accident: 6 (85%)	Majority of severe cases due to accidents	
	Moderate (GCS 12–9)	Fall: 1 (10%); Quarrel: 2 (20%); Accident: 7 (70%)	Accidents dominate moderate cases	< 0.001
	Mild (GCS 15–13)	Fall: 7 (13%); Quarrel: 9 (16%); Accident: 37 (69%)	Accidents also most frequent in mild cases	
Relationship between Trauma Type and Hearing Impairment	Fall	Normal: 6 (75%); CHL: 1 (12%); Mixed: 0; SNHL: 1 (12%)	Majority normal, few SNHL/CHL	< 0.05
	Conflict	Normal: 6 (50%); CHL: 2 (16%); Mixed: 2 (16%); SNHL: 2 (16%)	More varied impairments	
	Accident	Normal: 36 (72%); CHL: 9 (18%); Mixed: 1 (2%); SNHL: 4 (8%)	Normal most common, CHL notable	

GCS – Glasgow Coma Scale; CHL – Conductive Hearing Loss; SNHL – Sensorineural Hearing Loss

Table 4 demonstrates the relationship between different types of brain injuries and hearing impairments. Epidural injuries showed a significant association with hearing loss, as 40% of patients developed sensorineural hearing loss (SNHL) (p<0.001). Subdural injuries were mostly normal (75%), though 25% presented with mixed hearing loss. Contusions had the highest proportion of normal hearing (79%), but 14% developed conductive hearing loss (CHL), and small percentages showed mixed or SNHL. Brain edema cases were predominantly normal (66%), with 34% experiencing mixed loss. Temporal linear fractures revealed a more balanced distribution, with 40% normal, 30% CHL, and 30% SNHL. Skull base fractures were entirely normal (100%), while subarachnoid hemorrhage (SAH) cases were evenly split across normal, CHL, and SNHL (33.3% each). Pneumocephalus was mostly normal (66%), with 34% showing CHL. Overall, the findings highlight that while many injuries result in normal hearing, specific trauma types—particularly epidural and temporal linear fractures—are more strongly associated with hearing impairments.

**Table 4:** Relationship between type of injury and hearing impairments.

Brain Injury Type	Normal (n, %)	CHL (n, %)	Mixed (n, %)	SNHL (n, %)	P-value
Epidural	3 (60%)	0 (0%)	0 (0%)	2 (40%)	
Subdural	3 (75%)	0 (0%)	1 (25%)	0 (0%)	
Contusion	27 (79%)	5 (14%)	1 (3%)	1 (3%)	
Brain Edema	2 (66%)	0 (0%)	1 (34%)	0 (0%)	<0.001
Temporal Linear Fx	4 (40%)	3 (30%)	0 (0%)	3 (30%)	
Skull Base Fx	2 (100%)	0 (0%)	0 (0%)	0 (0%)	
SAH	1 (33.3%)	1 (33.3%)	0 (0%)	1 (33.3%)	
Pneumocephalus	2 (66%)	1 (34%)	0 (0%)	0 (0%)	

TBI – Traumatic Brain Injury; ENT – Ear, Nose, and Throat (specialist); GCS – Glasgow Coma Scale; CHL – Conductive Hearing Loss; SNHL – Sensorineural Hearing Loss; SAH – Subarachnoid Hemorrhage; Fx – Fracture.

## Discussion

Our findings align with previous studies on TBI-related auditory complications. TBI has been consistently linked to auditory and cognitive complications, with multiple studies highlighting diverse manifestations and risk factors. Kim et al. (2018) reported a case of auditory verbal agnosia in a 65‑year‑old patient, demonstrating how TBI can impair comprehension of spoken language while sparing recognition of environmental sounds and written language. This case underscores the importance of assessing auditory, cognitive, and language functions together, as auditory agnosia may be misdiagnosed as hearing loss or cognitive impairment.^20^

Hoover et al. (2017) examined speech perception in noise among TBI patients, finding that 84% experienced significant difficulty compared to only 9.1% of controls. Although cognitive measures such as attention and working memory were evaluated, no significant differences emerged, suggesting that TBI itself contributes to deficits in speech perception under challenging listening conditions.^14^ Similarly, Shangkuan et al. (2017) showed through a decade‑long cohort study that TBI patients are at higher risk of long‑term hearing loss, particularly those injured in traffic accidents or by penetrating trauma.^21^

Military populations have been a focus of research due to blast injuries. Karch et al.^22^ found that blast-exposed personnel had higher rates of significant threshold shifts (STS) and tinnitus compared to non-blast injuries, with tinnitus prevalence reaching 59% versus 40% (p <0.001). Gallun et al.^23^ further emphasized that veterans frequently report hearing loss, supported by behavioral and electrophysiological data. Both peripheral and central lesions have been documented, though diagnostic challenges remain due to limited imaging technologies for closed head injuries. Rehabilitation strategies such as hearing aids and auditory training show promise but require further validation.

Beyond auditory deficits, Rigon et al.^24^ highlighted gender differences in emotional cognition after TBI. Women outperformed men in both static and dynamic emotion recognition tasks, suggesting gender may act as a protective factor against social dysfunction. These findings expand the scope of TBI research beyond hearing loss to include psychosocial outcomes.

Other studies reinforce the multifactorial nature of hearing impairment after TBI. Lasak^25^ noted that hearing loss can occur at any point along the auditory pathway, affecting communication across all age groups. A research described how head trauma, including blast exposure, can cause irreversible damage to the basilar membrane and auditory nerve, with 10–24% of patients losing auditory nerve function.^26^ Munjal et al.^27^ confirmed that lower GCS scores correlate with more severe hearing impairment, consistent with findings in the present study. Temporal bone fractures, in particular, were strongly associated with cochlear damage and sensorineural loss, aligning with Aguilar et al.^28^

In addition to auditory and cognitive outcomes, the management of temporal bone fractures following TBI warrants consideration. In general, clinical practic, most temporal bone fractures do not require immediate neurosurgical intervention and are initially managed conservatively unless complicated by factors such as significant cerebrospinal fluid (CSF) leaks, facial nerve disruption, major vascular injury, or gross bone displacement identified on imaging. Conservative management typically includes stabilization, observation, and addressing complications such as CSF leaks with bed rest, head elevation, and, if necessary, lumbar drainage; most posttraumatic CSF leaks resolve spontaneously within several days to a week without surgery. Surgical intervention may be considered when conservative measures fail or in the presence of severe complications such as persistent CSF fistulae, complete facial nerve paralysis with electrodiagnostic evidence of degeneration, or other indications of structural disruption. Thus, the need for neurosurgical intervention in temporal bone fractures is relatively uncommon and is guided by the severity of associated injuries and clinical course rather than by the presence of the fracture alone.^29^

Finally, age-related hearing loss^30^ remains an important confounder, as senility contributes to bilateral, symmetrical deficits due to cochlear degeneration, cumulative noise exposure, and ototoxic medications. Taken together, these studies demonstrate that hearing impairment following TBI is influenced by injury mechanism, severity, anatomical site, and patient demographics. Rehabilitation requires a multidisciplinary approach, integrating audiological, cognitive, and psychosocial interventions to improve patient outcomes.

**Limitations**

Incomplete information on patient records in the hospital archives and the lack of cooperation of patients and their families in case of telephone calls to complete the desired checklists. Another limitation of this study is that because the study was conducted by reviewing files from previous years, the researchers did not have access to audiometric findings 1 month and 6 months after hearing injury and only had access to audiometric data after trauma and one year after trauma.

## Conclusion

This study of 70 patients with traumatic brain injury (TBI) and hearing impairment revealed that accidents were the predominant cause of trauma (71%), with men comprising the majority of cases (84%). Hearing complications such as otorrhagia and otorrhea were frequent, and right-sided involvement was most common. Statistical analysis demonstrated a significant change in hearing impairment over time (p = 0.03), indicating that auditory deficits persist or evolve during follow-up. Accidents were strongly associated with severe reductions in consciousness (GCS 8–3, p < 0.001) and remained the leading cause across all GCS levels. Furthermore, specific injury types, particularly epidural hematomas and temporal linear fractures, showed strong associations with sensorineural and conductive hearing loss, while other injuries such as contusions and subdural hematomas produced mixed outcomes. Overall, the findings highlight that trauma severity, mechanism, and anatomical site are critical determinants of hearing impairment in TBI patients.

The results emphasize the importance of early and comprehensive audiological evaluation in patients with TBI, especially those with accident-related injuries and temporal bone fractures. Since hearing impairment may persist or worsen over time, routine follow-up audiometry should be integrated into post-trauma care. Identifying the type of hearing loss (CHL vs. SNHL) is essential for tailoring rehabilitation strategies, including hearing aids, surgical interventions, or auditory training. Moreover, distinguishing auditory deficits from cognitive impairments is crucial to avoid misdiagnosis and ensure appropriate treatment. Clinicians should be aware that even patients with mild or moderate GCS scores may develop significant hearing complications, underscoring the need for vigilance across all trauma severities.

Future research should focus on larger, multicenter studies to validate these findings and explore long-term outcomes of hearing impairment in TBI patients. Advanced imaging and electrophysiological techniques could improve diagnostic accuracy, particularly for closed head injuries where subtle auditory pathway damage may be overlooked. Investigations into gender differences, psychosocial impacts, and rehabilitation efficacy (e.g., auditory training programs, assistive listening devices) would provide valuable insights. Preventive strategies, especially targeting traffic accidents, remain critical to reducing the burden of TBI-related hearing loss. Ultimately, integrating audiological care into trauma management protocols can enhance patient recovery, quality of life, and reduce long-term disability.

**Acknowledgements**

This article partially fulfills the requirements for the degree of general medicine by Mohammad Abdullahzadeh at Kermanshah University of Medical Sciences, Kermanshah, Iran.

**Authors’ Contribution**

R.F. and M.A. designed the study. M.A. participated in data collection. R.F. and M.S. analyzed and interpreted the data. R.F. supervised all the work. M.S. prepared the draft, and R.F. and M.A. revised it. All authors re-viewed and approved the final version.
